# Treatment of pancreatic ductal adenocarcinoma with tumor antigen specific-targeted delivery of paclitaxel loaded PLGA nanoparticles

**DOI:** 10.1186/s12885-018-4393-7

**Published:** 2018-04-23

**Authors:** Shu-ta Wu, Anthony J. Fowler, Corey B. Garmon, Adam B. Fessler, Joshua D. Ogle, Kajal R. Grover, Bailey C. Allen, Chandra D. Williams, Ru Zhou, Mahboubeh Yazdanifar, Craig A. Ogle, Pinku Mukherjee

**Affiliations:** 1 Department of Biological Sciences, University of North Carolina at Charlotte, Charlotte, NC 28223 USA; 2 Department of Chemistry, University of North Carolina at Charlotte, Charlotte, NC 28223 USA; 3 Department of Animal Laboratory Resources, University of North Carolina at Charlotte, Charlotte, NC 28223 USA

**Keywords:** PLGA, Nanoparticles, Mucin 1, Anti-MUC1 antibody, Targeted delivery, Paclitaxel, Pancreatic ductal adenocarcinoma

## Abstract

**Background:**

Pancreatic ductal adenocarcinoma (PDA) remains the most aggressive cancers with a 5-year survival below 10%. Systemic delivery of chemotherapy drugs has severe side effects in patients with PDA and does not significantly improve overall survival rate. It is highly desirable to advance the therapeutic efficacy of chemotherapeutic drugs by targeting their delivery and increasing accumulation at the tumor site. MUC1 is a membrane-tethered glycoprotein that is aberrantly overexpressed in > 80% of PDA thus making it an attractive antigenic target.

**Methods:**

Poly lactic-co-glycolic acid nanoparticles (PLGA NPs) conjugated to a tumor specific MUC1 antibody, TAB004, was used as a nanocarrier for targeted delivery into human PDA cell lines in vitro and in PDA tumors in vivo. The PLGA NPs were loaded with fluorescent imaging agents, fluorescein diacetate (FDA) and Nile Red (NR) or isocyanine green (ICG) for in vitro and in vivo imaging respectively or with a chemotherapeutic drug, paclitaxel (PTX) for in vitro cytotoxicity assays. Confocal microscopy was used to visualize internalization of the nanocarrier in vitro in PDA cells with high and low MUC1 expression. The in vivo imaging system (IVIS) was used to visualize in vivo tumor targeting of the nanocarrier. MTT (3-(4,5-Dimethylthiazol-2-yl)-2,5-Diphenyltetrazolium Bromide) assay was used to determine in vitro cell survival of cells treated with PTX-loaded nanocarrier. One-sided t-test comparing treatment groups at each concentration and two-way ANOVAs comparing internalization of antibody and PLGA nanoparticles.

**Results:**

In vitro, TAB004-conjugated ICG-nanocarriers were significantly better at internalizing in PDA cells than its non-conjugated counterpart. Similarly, TAB004-conjugated PTX-nanocarriers were significantly more cytotoxic in vitro against PDA cells than its non-conjugated counterpart. In vivo, TAB004-conjugated ICG-nanocarriers showed increased accumulation in the PDA tumor compared to the non-conjugated nanocarrier while sparing normal organs.

**Conclusions:**

The study provides promising data for future development of a novel MUC1-targeted nanocarrier for direct delivery of imaging agents or drugs into the tumor microenvironment.

**Electronic supplementary material:**

The online version of this article (10.1186/s12885-018-4393-7) contains supplementary material, which is available to authorized users.

## Background

Pancreatic Cancer is a highly aggressive disease with a 5-year relative survival rate of ~ 9% [1]. Greater than 90% of all pancreatic cancers arise in the epithelial ducts of the pancreas and are designated pancreatic ductal adenocarcinomas (PDA). Only 18-20% of patients diagnosed with PDA are eligible for surgical resection followed by chemo and radiation therapies. For majority of PDA patients, chemo and radiation therapies are the only choices. However, due to chemo-resistance, the overall survival rate with or without surgical resection remains dismal [2]. It is established that one of the reasons for failed therapy is the inefficient delivery of chemotherapy drugs to the tumor site, likely due to the dense stroma and deficient vascular network in the pancreatic tissue microenvironment [3, 4]. Therefore, there is a pressing need to develop a novel drug delivery system for PDA that can increase the drug accumulation and uptake in a tumor specific manner [5].

Nanoparticles (NPs) modified to degrade in the tumor microenvironment or target tumor antigens are promising platforms for the targeted delivery of therapeutic drugs to specific cells and tissues [6–9]. NPs formulated from biodegradable and biocompatible polymers, such as Poly lactic-co-glycolic acid (PLGA), are being utilized increasingly in research due to their excellent systemic characteristics [10]. PLGA NPs allow for the encapsulation of a variety of hydrophobic chemotherapeutics or imaging agents, and can thereby facilitate the systemic delivery of these otherwise insoluble compounds with localization at the tumor site. This localization is the result of the enhanced permeability and retention effect (EPR), which is caused by the vasculature permeability in tumors being greater than in normal tissues, and thus provide a mechanism of selection for the NPs, as they do not penetrate into neighboring normal tissue [11, 12]. The unorganized structure of the tumor and lack of lymphatic drainage prolong the retention of NPs after they escape from the leaky vasculature [13]. PLGA NPs with polyethylene glycol (PEG) displayed at the surface have been shown to increase circulatory half-lives of the NPs, while surface modification with targeting agents have been shown to aid in localization of the NPs selectively at targeted tissues [14–17]. Novel chemotherapeutic agents and combinations like FOLFIRINOX (5-fluorouracil, oxaliplatin, irinotecan, and leucovorin) or Abraxane (nab-paclitaxel, an albumin-coated formulation of paclitaxel) have been developed and have seen some success [18, 19]. The combination of gemcitabine and nab-paclitaxel has been shown to increase the intratumor concentration of gemcitabine by roughly three-fold in xenograft models [20, 21]. Paclitaxel, a taxane agent, (PTX) is one of the most widely used anticancer drugs approved for the treatment of many types of cancer. PTX interferes with cell division by interacting with the polymer form of tubulin and promoting microtubulin assembly. This stabilizes the polymers against depolymerization, which induces M-phase cell cycle arrest and cell death [22, 23]. Targeted NPs consisting of PLGA encapsulated PTX will provide a drug delivery system that would increase delivery of PTX to the tumor site, due to the EPR effect [24]. Systemic administration of the drug loaded NPs, however, have many problems associated with it. For instance, if the NP is too large, issues can arise that prevent them from reaching the tumor site, as the NPs have to cross through several biological barriers, such as blood vessels, tissues, organs, and cells. Without any specificity for the tumor site, it may be necessary to use fairly high doses of NPs and drugs to achieve sufficient local concentrations. In PDA, due to poor vascularization and despoplasia, non-targeted NPs may not suitable. Conjugating the NPs with tumor targeting moieties could possibly overcome some of these challenges.

Mucin-1 (MUC1), is a transmembrane protein with an extracellular domain that is heavily glycosylated [25]. It is normally expressed on epithelial cells of the mammary gland, esophagus, stomach, duodenum, uterus, prostate, lung, and pancreas [26]. In healthy tissues, the negatively charged glycosylated extracellular domain of MUC1 creates a physical barrier and an anti-adhesive surface, preventing pathogenic colonization [27]. In over 80% of PDA, MUC1 is hypoglycosylated and overexpressed [28] which in turn is also associated with higher metastasis and poor prognosis [29, 30]. These characteristics ranked MUC1 as one of the best tumor antigens for targeted therapy [31]. We have developed a novel monoclonal antibody, TAB004 (OncoTAb, Inc., Charlotte, NC), which specifically targets the hypoglycosylated form MUC1 (tMUC1) [32–34].

This study was aimed at investigating the targeting ability of TAB004 conjugated PLGA NPs in vitro and in vivo. NR, FDA, and ICG were used as the imaging agents and PTX as the chemotherapeutic drug. We hypothesized that the conjugation of TAB004 to the surface of PLGA NPs will increase their accumulation and duration at the tumor site and thereby increase the overall therapeutic index of the treatment. For this purpose, PTX, ICG, or FDA&NR were encapsulated in PEGylated PLGA NPs and then conjugated to TAB004. Unconjugated particles were used as controls. Internalization, retention, and therapeutic efficacy were evaluated in vitro in several MUC1 high and low expressing human PDA cell lines.

## Methods

### Materials

All chemical reagents used for the study were of analytical grade. Poly(DL-lactide-co-glycolide) M_W_ 20,000 (PLGA) (50:50) PolySciences, Inc. (Warrington, PA). Polyethylene glycol M_w_ 1000 (PEG_1000_), Poly(vinyl alcohol) M_W_ 6000 (PVA) (80 mol% hydrolyzed), 1,1′-carbonyldiimidazole (CDI), 1,3-diaminopropane (DAP), Dextrose were purchased from Sigma Aldrich (St. Louis, MO). Paclitaxel was purchased from Matrix Scientific (Columbia, SC), ICG was purchased from Chem-Impex (Wood Dale, IL). TAB004 monoclonal antibody was obtained from OncoTAb, Inc. (Charlotte, NC, USA).

### Cell culture

Human PDA cell lines including BxPC3, HPAC, HPAFII, and MIA PaCa-2 were purchased from ATCC (Manassas, VA). Murine PDA cell line, KCM was generated in our lab [35]. KCM, HPAC, HPAF II, and MIA PaCa-2 were maintained in Dulbecco’s modified Eagle’s medium (DMEM, 11965-092, Gibco). BxPC3 cell lines were maintained in RPMI medium 1640 (RPMI, 11875-093, Gibco). Growth media for these cell lines were supplemented with 10% fetal bovine serum (FBS, Gibco), 3.4 mM ˪-glutamine, 90 units (U) per ml penicillin, and 90 μg/ml streptomycin (Cellgro).

### Determination of NP loading

For the paclitaxel NP (PTX) formulation a 20 mg sample of was dissolved into 600 μl of DMSO-*d*6 and the concentration of the respective cargo determined using ^1^H NMR at 25 °C by comparing unique resonances of the cargo to the methylene residue of PLGA at [5.2 ppm]. For the fluorescein diacetate (FDA), indocyanine green (ICG), and Nile red (NR) NP formulations, a sample of nanomaterial (2-4 mg) was dissolved into DMSO and the amount of cargo quantified by UV-Vis.

$$ Encapsulation\ Efficiency=\frac{Amount\ of\ cargo\ encapsulated}{Amount\ of\ cargo\ used}\times 100 $$

### Determination of NP size and polydispersity

Particle size, polydispersity index (PDI), along with zeta potential were determined by dynamic light scattering (Zetasizer Nano, Malvern Instruments) Table 1.

**Table 1:** Structural Properties of PTX, FDA, NR, and ICG Nanomaterials

Nanomaterial	HD (nm)	PDI	ZP (ζ)
PTX NPs	141.8 ± .6	.155 ± .009	−7.6 ± .2
FDA NPs	171.3 ± 1.2	.129 ± .004	−6.4 ± .2
NR NPs	209.3 ± .6	.175 ± .007	−6.7 ± .1
ICG NPs	180.7 ± .9	.076 ± .006	−6.8 ± .1

*HD* Hydrodynamic Diameter, *PDI* Polydispersity Index, *ZP* Zeta Potential

### Cargo release profiles

Release profiles of NPs were modeled using FDA NPs. The release characteristics of these particles were characterized in phosphate buffered saline (PBS) at pH 7.4.

### Synthesis of PCL_14K_-PEG_1000_

PCL_14K_-PEG_1000_ was prepared according to the following procedure. Polycaprolactone (2 g, M_w_ ~ 14,000) was added to a 50 ml oven dried round-bottom flask fitted with a claisen adapter and equipped with a magnetic stir bar, a rubber septum, and a reflux condenser with attached drying tube. To this was added 20 ml of thionyl chloride via syringe, and the rubber septum replaced with a ground-glass stopper, and the resulting solution heated to reflux for 3 h. The thionyl chloride was then removed under reduced pressure using a rotary evaporator. The resulting residue was placed under a nitrogen atmosphere and 50 ml of freshly distilled tetrahydrofuran (THF) was added by cannula followed by PEG_1000_-diol (2.9 g, 20 equivalent) and triethylamine (2 ml, 14.35 mmol). The resulting solution was left to stir for 18 h at room temperature. This solution was then poured into 500 ml of DI water under vigorous stirring to precipitate the desired product and remove unreacted PEG_1000_ diol. The precipitate was isolated by filtration, re-dissolved into THF (50 ml), and precipitated as before. This process was repeated three times. Finally, the isolated product was dried under vacuum at 25 °C for 72 h. The desired product was isolated as a solid white material (1.13 g, 53%). ^1^H NMR (500 MHz CDCl_3_): δ 1.36 (m, -CH_2_CH_2_CH_2_-), 1.63 (m, -CH_2_CH_2_CH_2_-), 2.28 (t, -C(O)CH_2_-), 3.62 (s, -OCH_2_CH_2_-), 4.04 (t, -OCH_2_).

### Synthesis of PCL_14K_ -PEG_1000_-NH2

PCL_14K_ -PEG_1000_-NH2 was prepared by the according to the following procedure. PCL_14K_ -PEG_1000_ (1 g) was added to a 50 ml oven dried 2-neck round-bottom flask equipped with a magnet stir bar and a rubber septum with nitrogen inlet. To this was added 20 ml of dry methylene chloride (DCM) followed by 1,1′-carbonyldiimidazole (100 mg, .62 mmol) and the resulting solution left to stir for 6 h at room temperature. To this was added 1,3-diaminopropane (1 ml, 12.19 mmol) and the resulting solution left to stir for 12 h at room temperature. The DCM was then removed under reduced pressure using a rotary evaporator. The resulting viscous yellow liquid was dissolved into THF (20 ml) and precipitated by pouring the solution into 250 ml of vigorously stirred DI water. The precipitate was isolated by filtration, re-dissolved into THF (20 ml), and precipitated as before. This process was repeated three times. Finally, the isolated product was dried under vacuum at 25 °C for 72 h. The desired product was isolated as a yellow solid (.5 g, 50%). Although the resonances for the end-group -C(O)NHCH_2_CH_2_CH_2_NH_2_- are not assigned due to obfuscation of these resonances by the polymer backbone, the polymer tested positive for the presence of primary amines using the Kaiser test [36]. H NMR (500 MHz CDCl_3_): δ 1.37 (m, -CH_2_CH_2_CH_2_-), 1.64 (m, -CH_2_CH_2_CH_2_-), 2.29 (t, -C(O)CH_2_-), 3.63 (s, -OCH_2_CH_2_-), 4.05 (t, -OCH_2_).

### Nanoparticle preparation: General method

Nanoparticles (NPs) were prepared by the nanoprecipitation according to the method of Langer et al. [37]. Briefly; 100 mg of PLGA (50:50, M_w_ ~ 20 K), 5 mg of PCL-PEG_1000_, 1 mg PCL-PEG_1000_-NH2, and 1 - 5 mg of cargo was dissolved into 10 ml of acetone. This solution was then added dropwise via syringe into a stirred solution of 1% PVA (20 ml) at a rate of 90 ml/hr. controlled using a syringe pump. The resulting colloidal suspension was then transferred to a 100 ml round-bottom flask, and the acetone removed under reduced pressure using a rotary evaporator. NPs were then purified by centrifugation (25 min, 30,000×g) using three successive washes of sterile filtered 18 Ω water at 4 °C. The resulting NP pellet was then resuspended into sterile filtered 18 Ω water (10 ml), whereupon dextrose (10 mg) was added as a lyoprotectant. This colloidal suspension was then flash frozen in liquid nitrogen then lyophilized at 25 °C and 50 mTorr for 24 - 48 h resulting in a flocculent solid. Paclitaxel (PTX), Fluorescein Diacetate (FDA), and Nile Red (NR) were all prepared according to the general method described above. See Table 2 for the amount of cargo used in the preparation of the respective nanomaterials.

**Table 2:** Cargo Loading of PTX, FDA, NR, and ICG into Nanomaterials

Nanomaterial	Cargo Loading (mg)	Amount Encapsulated (mg/100 mg NPs)	EE (%)
PTX NPs^a^	5	2.3 ± .1	46.7 ± 1.8
FDA NPs^b^	5	1.7 ± .1	34.2 ± .9
NR NPs^b^	2	.26 ± .01	13.1 ± .6
ICG NPs^b^	1.75	.39 ± .01	22.1 ± .3

*EE* Encapsulation Efficiency. ^a^Determined by 1H NMR. ^b^Determined by UV-Vis

### ICG preparation

ICG NP’s were prepared similarly using a modified nanoprecipitation method according to procedure reported by Cai [38]. Briefly; 100 mg of PLGA (50:50, M_w_ ~ 20 K), 5 mg of PCL-PEG_1000_, 1 mg PCL-PEG_1000_-NH2 was dissolved into 9 ml of acetonitrile. Meanwhile 1 mg of ICG was dissolved into 1 ml of sterile filtered 18 Ω water. The two solutions were then mixed, and vortexed rapidly for 2 min. The resulting solution was then added dropwise via syringe into a stirred solution of 1% PVA (20 ml) at a rate of 90 ml/hr. controlled using a syringe pump. The resulting colloidal suspension was then transferred to a 100 ml round-bottom flask, and the acetonitrile removed under reduced pressure using a rotary evaporator. NPs were then purified by centrifugation (25 min, 30,000×g) using three successive washes of sterile filtered 18 Ω water at 4 °C. The resulting NP pellet was then resuspended into sterile filtered 18 Ω water (10 ml), whereupon dextrose (10 mg) was added as a lyoprotectant. This colloidal suspension was then flash frozen in liquid nitrogen, and lyophilized at 25 °C and 50 mTorr for 24 - 48 h resulting in a flocculent green solid.

### FDA release profiles

1 ml Solutions of FDA NPs (1 mg/ml) in PBS (pH 7.4) were incubated with constant stirring at 37 °C over 72 h. 100 μl samples were taken at the indicated time-points and centrifuged (16,000 x g) to pellet out the remaining NPs. 50 μl of NP free buffer was removed carefully so as not to disturb the pellet, and 20 μl of 5% NaOH added to hydrolyze the liberated FDA. Absorbance measurements were recorded at 490 nm. These values were compared to a control sample having been dissolved in a 50:50 acetonitrile (CAN):H_2_O solution to liberate the entire sample of FDA from the NPs, and hydrolyzed as above.

### SEM imaging

Lyophilized nanoparticles were re-suspended in H_2_O at 0.01 mg/mL concentrations and sonicated for 10 s. Samples were placed on SPI 5 × 5 silicon chips and dried overnight at 40 °C. Scanning electron microscopy (SEM) images were obtained with a Raith 150 microscope operated at 10 kV.

### Generation of BxPC3.MUC1, BxPC3.Neo, and KCM-Luc

Full-length MUC1 gene was cloned into the pLNCX.1 vector consisting of the neomycin resistance gene for retroviral infection. GP2-293 cells were transfected with MUC1 pLNCX.1 and pVSV-G vectors and the resulting viral supernatant used to infect a MUC1-null human PDA cell line, BxPC3. These cells were designated BxPC3.MUC1 cells. BxPC3.MUC1 serves as MUC1-high positive control. BxPC3.Neo represent BxPC3 cells that express the empty vector and therefore serves as the MUC1-negative controls [30]. KCM cell line was generated from spontaneous PDA tumors arising in the PDA.MUC1 triple transgenic mice. This cell line is syngeneic to the C57/Bl6 mouse background and expresses human MUC1 [34, 35]. Retroviral transduction of KCM cells with MSCV Luciferase PGK-Hygro (MSCV Luciferase PGK-hygro was a gift from Scott Lowe, Addgene plasmid # 18782) was performed by transfecting GP2-293 cells with the MSCV Luciferase PGK-Hygro and pVSV-G vectors and using the subsequent viral supernatant to infect KCM cells.

### Conjugation of TAB004 to PLGA NPs

TAB004 conjugation to PLGA nanoparticles was performed using NuLink conjugation kit (NuChemie). PLGA nanoparticles were weighed out into an appropriate Eppendorf tube. To a vial containing 1 mg of the NuLink© bis-electrophile thioester was added 1 drop of DMSO to assist with dissolution, then 500 μl of 18 Ω H_2_O was added. The solution was vortexed until all of the labeling reagent was dissolved. The PLGA nanoparticles were re-suspended in 200 μl of 18 Ω H_2_O. The labeling solution was added dropwise to the nanoparticles while under a gentle vortex and allowed to incubate at room temperature (how long time) after mixing. The labeled nanoparticles were centrifuged at 21,000 rcf and 4 °C to pellet them. The supernatant was removed and the labeled nanoparticles re-suspended in 200 μl of 18 Ω H_2_O. 30 μg of TAB004 at mg/ml conc (in azide free buffer) was then added to the labeled NP solution in one portion. The next day, the nanoparticles were centrifuged at 21,000 rcf and 4 °C to pellet them and the supernatant discarded. Nanoparticles were re-suspended into desired working volume of PBS. Successful TAB004 conjugation to the PLGA nanoparticle was confirmed using FACS (BD Fortessa) and an anti-mouse IgG_1_-FITC secondary antibody.

### Cell viability assays

Cell viability assays were performed using MTT (3-(4,5-Dimethylthiazol-2-yl)-2,5-Diphenyltetrazolium Bromide) (Fisher Scientific, USA). Optimal number of cells per cell line were plated into 96-well tissue culture plates to ensure cells would not be over confluent after 48 h post treatment. 24 h after cells were plated, they were treated with corresponding concentrations of dimethyl sulfoxide (DMSO), PTX, blank PLGA nanoparticles, PTX loaded PLGA nanoparticles, and TAB004 conjugated PTX loaded PLGA nanoparticles for 1.5 h. After 1.5 h the treatments were washed off with 1× PBS and 200 μl of fresh media was added to the wells and cell lines were incubated for 48 h at 37 °C, > 90% humidity, and 5% CO_2_ conditions. Following the 48 h incubation, the media was replaced with 100 μl of phenol red free media and 10 μl of MTT was added to each well. Plates were incubated at 37 °C, > 90% humidity, and 5% CO_2_ conditions for 4 h, after which the media and MTT were removed, 100 μl of DMSO added, and incubated at 37 °C for 10 min. The plates were then read using a ThermoFisher Scientific MultiScan GO. Cell viability data for each treatment group (PTX, NP loaded with PTX, and TAB004 conjugated to NPs loaded with PTX) was normalized to their own vehicle control cell viabilities (DMSO and Blank NPs).

### Internalization of NPs

Cell lines were plated into 4-chamber well slides (154, 917, LAB-TEK) at optimal concentration to ensure cells would not be over confluent after 24 h. 24 h after cells were plated, they were treated with fluorescein (20 μg/ml), or fluorescein diacetate and Nile Red containing PLGA nanoparticles at 1 mg/ml concentration for 1.5 h at 37 °C, > 90% humidity, and 5% CO_2_ conditions. After treatment, cells were washed with PBS for 5 min (3×) and fixed with 4% formaldehyde. Prolong Gold Antifade reagent with DAPI (P36935, Molecular Probes) was applied to mount coverslips. Images were acquired on an Olympus Fluoview FV 1000 confocal microscope.

### Specificity and internalization of TAB004

TAB004 conjugation to pHrodo Red was performed using the pHrodo Red, succinimidyl ester (pHrodo Red, SE) kit (P36600, Molecular Probes). TAB004 conjugation to indocyanine green (ICG) was performed using the ICG Labeling Kit –NH_2_ (LK31-10, Dojindo Molecular Technologies, Inc.). All conjugations were performed using manufacturer protocols. Cell lines were plated into 4-chamber well slides (154,917, LAB-TEK) at optimal concentration to ensure cells would not be over confluent after 24 h. 24 h after cells were plated, they were treated with 5 μl of TAB004-phRodo Red conjugation solution for various time points at 37 °C, > 90% humidity, and 5% CO_2_ conditions. During the last 5 min of treatment, Wheat Germ Agglutinin-Alexa Fluor 488 conjugate (W11261, Molecular Probes), was added to each chamber at 5 μg/ml. The cells were washed with PBS for 5 min (3×) and fixed with 4% formaldehyde. Prolong Gold Antifade reagent with DAPI (P36935, Molecular Probes) was applied to mount coverslips. Images were acquired on a GE Healthcare Life Sciences DeltaVision Elite Imaging microscope.

### Mouse strains

C57Bl/6 mice were purchased from Jackson Laboratory and housed at UNC Charlotte’s vivarium.

### Orthotopic tumor model

C57/Bl6 female mice were injected in the pancreas with 5 × 10^5^ KCM-Luc cells and allowed to recuperate for 7 days before any experiments were performed. This study and all procedures were performed after approval from the Institutional Animal Care and Use Committee of UNC Charlotte.

### Visualization of KCM-Luc orthotopic tumors, TAB004-ICG, and TAB004 conjugated PLGA NPs with ICG

Orthotopic KCM-Luc tumor bearing C57/Bl6 mice were injected with 125 μl of Redijet D-Luciferin (760,504, Perkin Elmer) intraperitoneally and imaged 25 min later with a Perkin Elmer IVIS Spectrum. Orthotopic KCM-Luc tumor bearing C57/Bl6 mice were injected with 25 μg of TAB004-ICG, 50 mg/kg of NP w/ICG, or 50 mg/kg of TAB004-NP w/ICG intraperitoneally and imaged at various time points with a Perkin Elmer IVIS Spectrum. Mice were euthanized at the end of imaging studies. All procedures were conducted in accordance to the Institutional Animal Care and Use Committee of UNC Charlotte. All mice and organ images and region of interests (ROIs) were acquired and processed in Living Imagine 4.3.1 (Caliper Life Sciences, Waltham, MA).

## Results

### Nanoparticle preparation and characterization

**Fig. 1:**
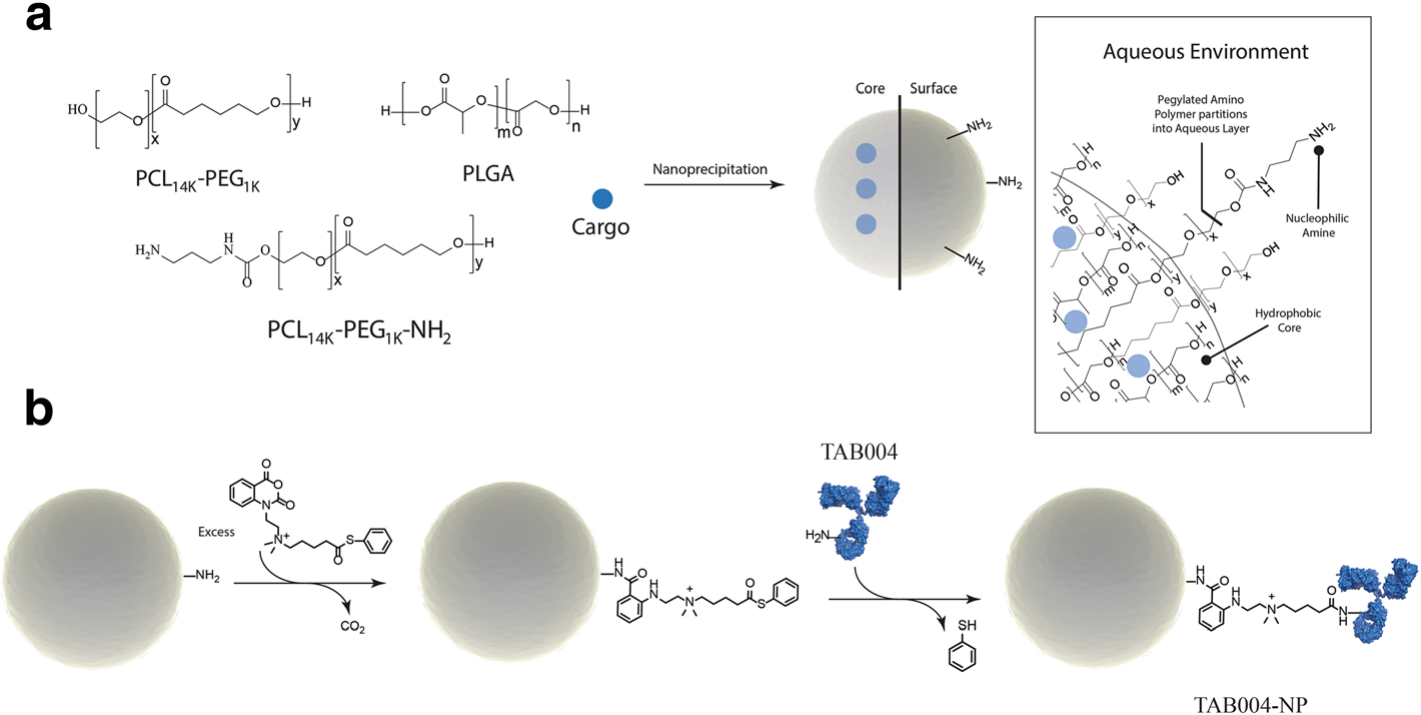
Nanoparticle preparation and surface functionalization. **a** Nanoparticle preparation by nanoprecipitation; (**b**) Functionalization of the NP surface using the NuLink bis-electrophile

We evaluated the size and release profile of PLGA NPs to determine an optimal size for use (Fig. 1a). As shown, PCL_14K_-PEG_1K_ and PCL_14K_-PEG_1K_-NH_2_ partitions into the aqueous environment during self-assembly of the nanoparticles, thereby generating a nanoparticle having a pegylated surface with a small percentage of nucleophilic amines available for chemical modification. During self-assembly the cargo is encapsulated in the hydrophobic core. The functionalization of the NP surface was performed using the NuLink bis-electrophile (Fig. 1b).

**Fig. 2:**
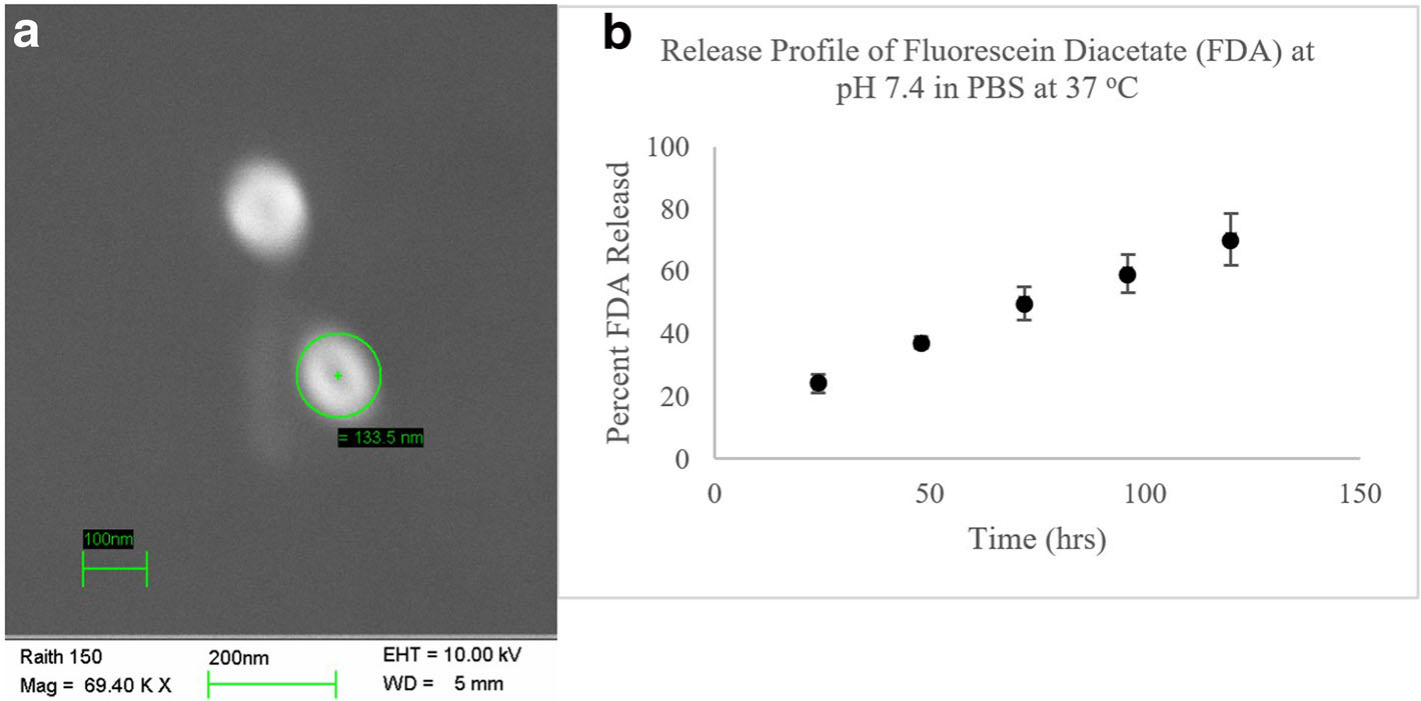
Characterization of PLGA NPs: Fluorescein Diacetate (FDA) PLGA NPs were used as a model system to investigate the size and release profile of the NP platform described (**a**, **b**). In vitro cargo release of the NPs was evaluated in PBS at pH 7.4. FDA was steadily released over the course of 120 h. The percent of FDA released at 24, 48, 72, and 96 h was 24%, 37%, 50%, 59%, and 70% respectively (**b**)

Fluorescein Diacetate (FDA) PLGA NPs were used as a model system to investigate the size and release profile of the NP platform described (Fig. 2a, b). In vitro cargo release of the NPs was evaluated in PBS at pH 7.4. FDA was steadily released over the course of 120 h. The percent of FDA released at 24, 48, 72, and 96 h was 24%, 37%, 50%, 59%, and 70% respectively (Fig. 2b).

### PLGA NPs internalize into BxPC3.MUC1 and BxPC3.Neo human PDA cell lines

**Fig. 3:**
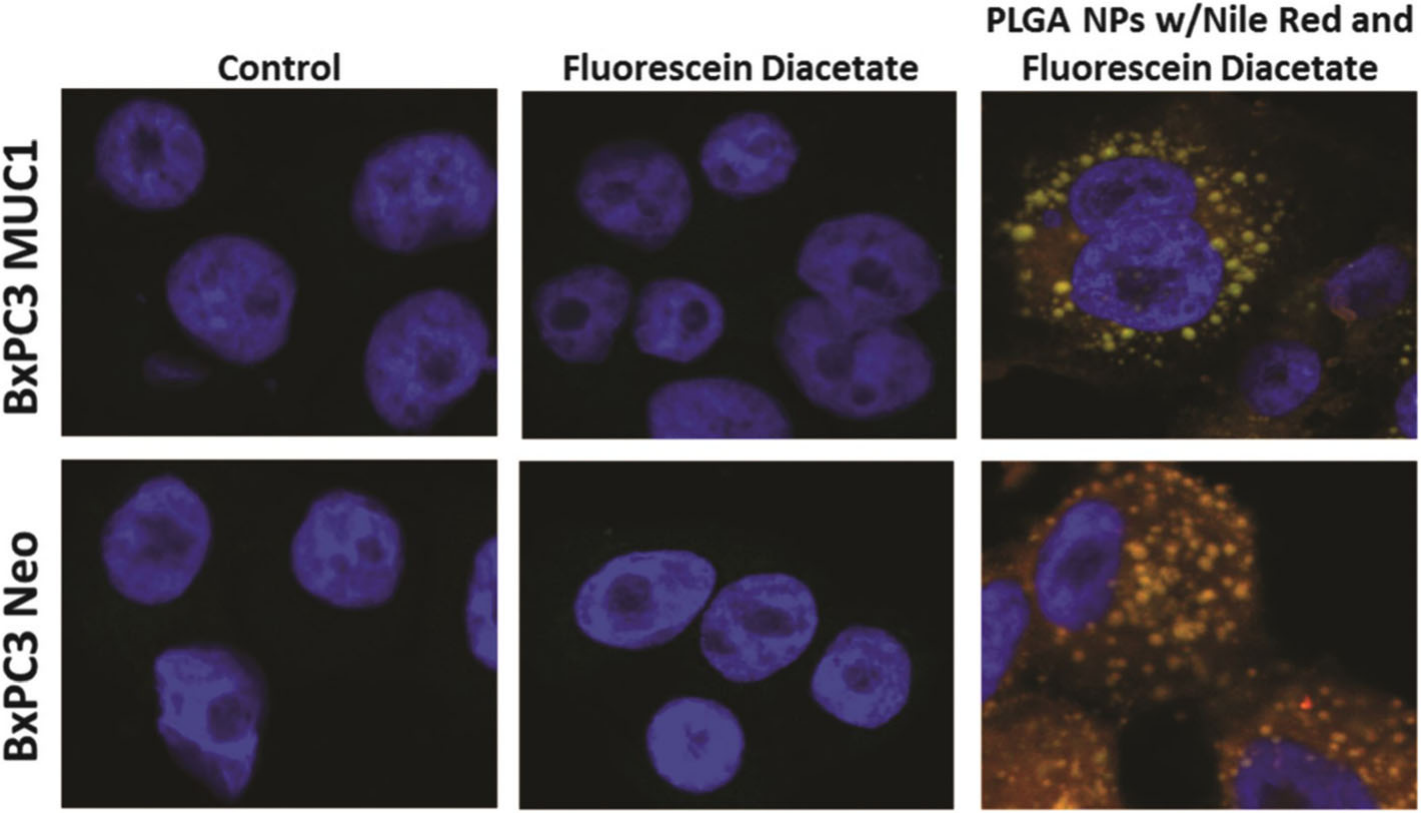
Internalization of PLGA NPs in: (A) BxPC3 MUC1; (B) BxPC3 Neo. Results shown are representative images (*n* = 3)

We determined whether the PLGA NPs internalizes into a human PDA cell line. Wild-type BxPC3 cells have minimal expression of endogenous MUC1. We generated BxPC3.MUC1 cells that stably express full-length MUC1. As control, we generated BxPC3.Neo that expresses the empty vector. BxPC3.MUC1 cells express high levels of MUC1 while BxPC3.Neo cells express minimal levels of MUC1 [30]. In the later experiments, this will enable us to assess the specificity of the TAB004 antibody in an otherwise genetically identical PDA cell line. BxPC3.MUC1 and BxPC3.Neo cell lines were treated for 1.5 h with FDA and Nile Red loaded NPs. This matched the total treatment time of PDA cell lines in the cell viability assay. After 1.5 h, FDA and NR loaded PLGA NPs internalized through endocytosis into both BxPC3.MUC1 and BxPC3.Neo cell lines equally (Fig. 3) suggesting that internalization is independent of MUC1 expression levels. Punctate green fluorescence, from the hydrolysis of FDA in the NPs, can be seen within the cytoplasm of the cells, indicating internalization of the NPs (Fig. 3). Fluorescence is only observed if FDA is hydrolyzed within the cells [39]. Although we detect a slight trend of increased endocytosis in the BxPC3.MUC1 versus BxPC3.Neo cells, the difference is not statistically significant.

### TAB004 antibody internalizes into PDA cell lines that express tMUC1

**Fig. 4:**
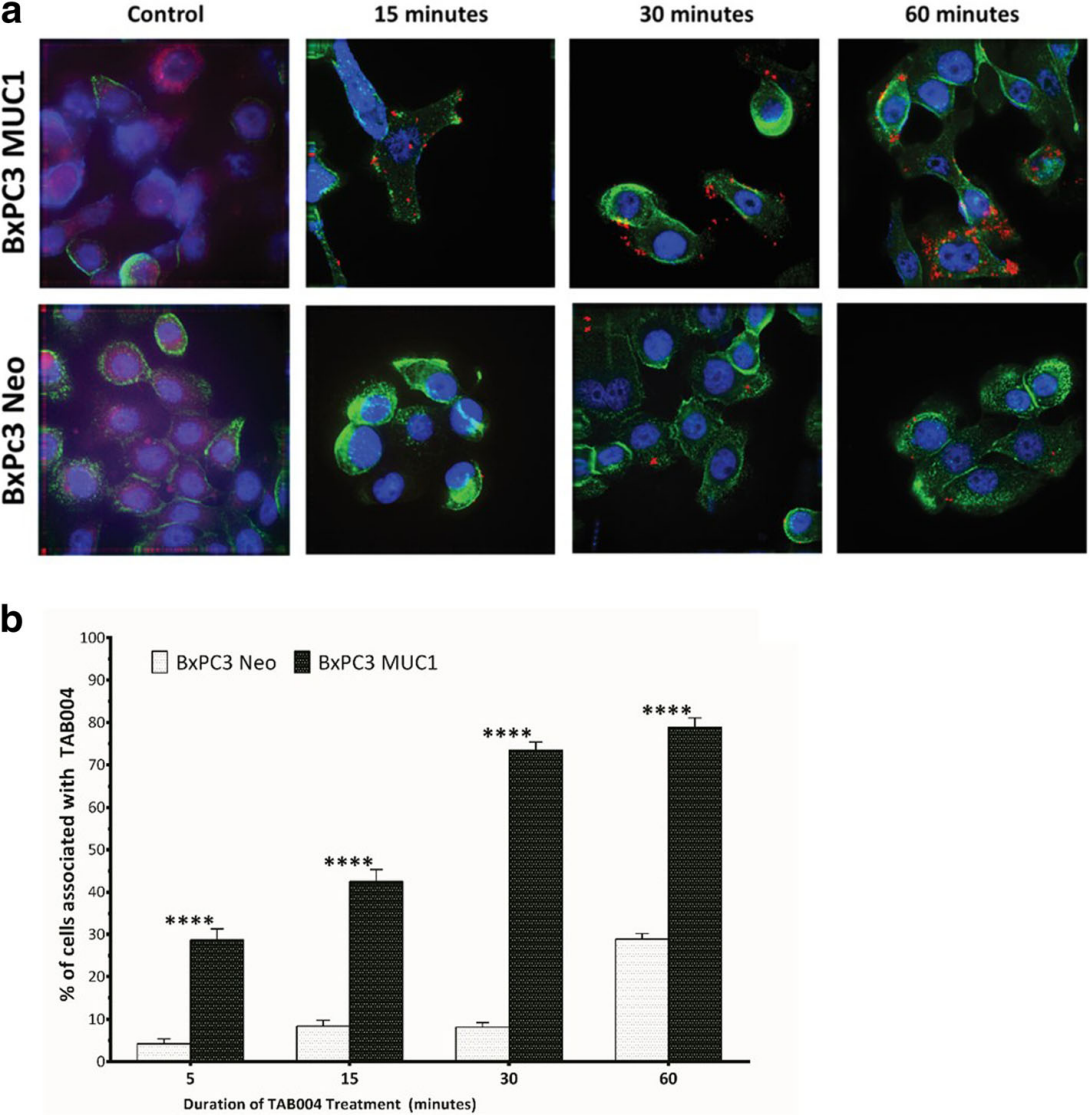
Specificity and internalization of TAB004 in BxPC3 MUC1 and Neo: (**a**) representative images cells treated with TAB004 conjugated to pHRodo red (*n* = 3); (**b**) quantification of images determine by the number of cells associated with TAB004 divided by total number of cells. Data shown is mean ± SEM (*n* = 3) and determine by two-way ANOVA and Bonferroni’s post-hoc test, *p < 0.05, **p < 0.01, ***p < 0.001, ****p < 0.0001

Next we determined the specificity of TAB004 to MUC1 and quantified the internalization of TAB004 antibody by fluorescent microscopy (Fig. 4). The presence and uptake of TAB004 was visualized by conjugating the antibody to pHrodo Red, which is non-fluorescent outside the cell, but fluoresces red only post endocytosis (Fig. 4a). The green fluorescence is wheat germ agglutinin that stains the cell membrane. The fluorescent signal from TAB004 is significantly increased in BxPC3.MUC1 when compared to BxPC3.Neo cells at all time points (Fig. 4b). There is some internalization observed in BxPC3 Neo, which can be caused by the very low level of endogenous MUC1 that is present, or by non-specific endocytosis as PDA cells have been shown to actively swallow their surroundings through macropinocytosis [40, 41].

### TAB004 conjugated PTX loaded PLGA NPs are specific for and inhibit growth of tMUC1 expressing cells

The successful conjugation of TAB004 to the surface of the NPs (T-NPs) was determined by flow cytometry (Additional file 1: Figure S1). The linking reagent, a thioester, was tested and was successful in linking TAB004 to the NPs (Additional file 1: Figure S1B). Flow cytometry data shows a shift in fluorescence when NPs are conjugated to TAB004 and labeled with FITC conjugated anti-mouse IgG1. Unconjugated NPs did not display any shift in fluorescence (Additional file 1: Figure S1A). NPs without the linking reagent but incubated with TAB004, or FITC anti-mouse IgG1, or both also served as controls and as expected did not show any shift in fluorescence signal. This suggests that the thioester linker was successful in conjugating TAB004 to the NPs.

**Fig. 5:**
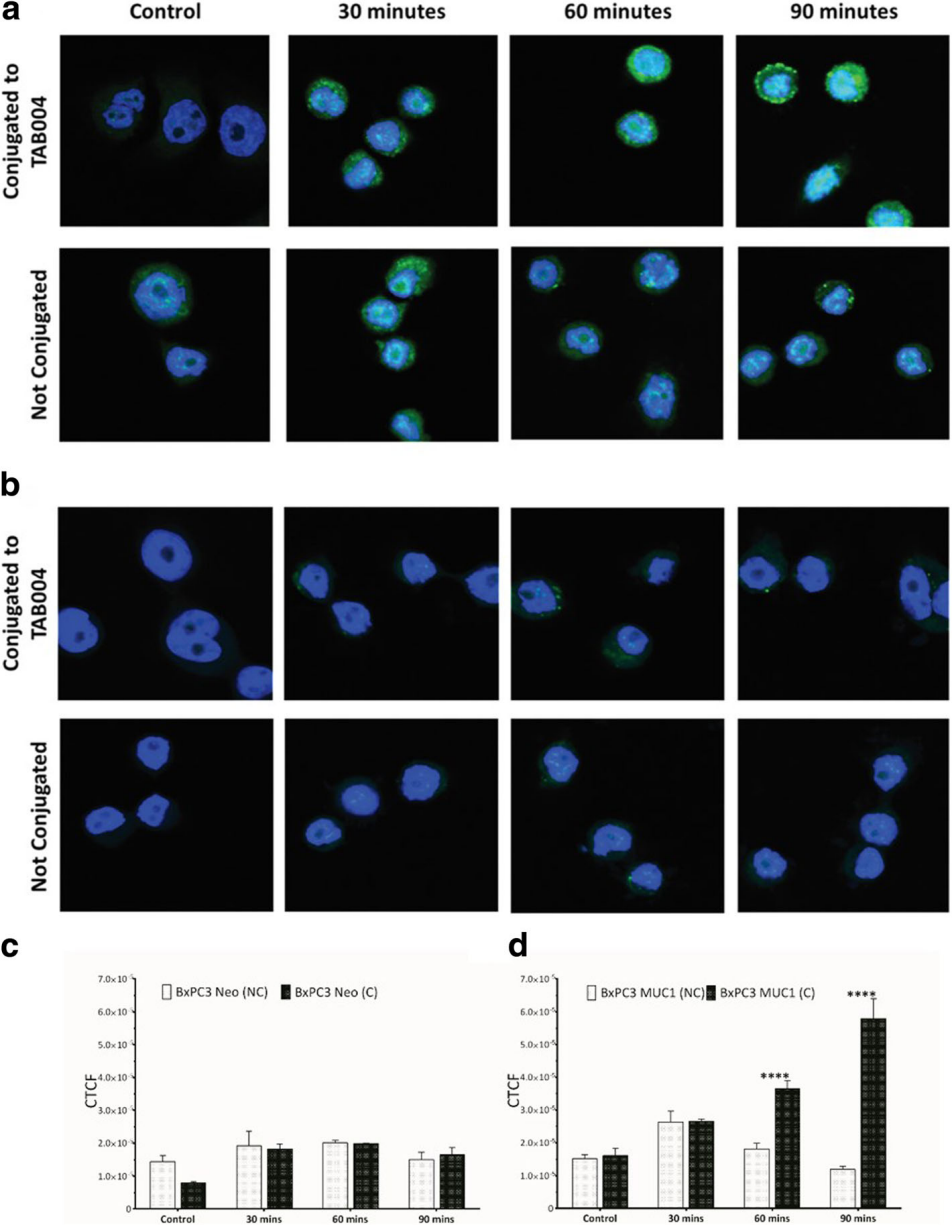
Internalization of TAB004 conjugated NPs loaded with fluorescein diacetate (FDA): (**a**) representative images of BxPC3 MUC 1 (*n* = 3) treated with T-NPs; (**b**) representative images of BxPC3 Neo (*n* = 3) treated with T-NPs; (**c**) and (**d**) quantification of fluorescence using Image J to determine corrected total cell fluorescence (CTCF). Data shown is mean ± SEM (*n* = 3) and determined by a one-sided t-test, *p < 0.05, **p < 0.01, ***p < 0.001, ****p < 0.0001

We compared the internalization of unconjugated NPs to TAB004 conjugated NPs (T-NPs) in MUC1 high BxPC3.MUC1 versus MUC1 low BxPC3.Neo cells. BxPC3.MUC1 and BxPC3.Neo cells were treated with FDA loaded NPs or T-NPs and fluorescence signal quantified over time (Fig. 5). There was no significant increase in fluorescence between T-NPs and NPs in the MUC1-low BxPC3.Neo cells (Fig. 5c). However, a significant increase in fluorescence was detected in BxPC3.MUC1 cells treated with T-NPs compared to when treated with NPs. This significant increase in fluorescence was observed at 60 and 90 min post treatment. The data indicates that linking TAB004 to the NPs was highly effective in longer term retention of the NPs within the MUC1-high cells compared to NPs alone and that this retention was antigen specific.

**Fig. 6:**
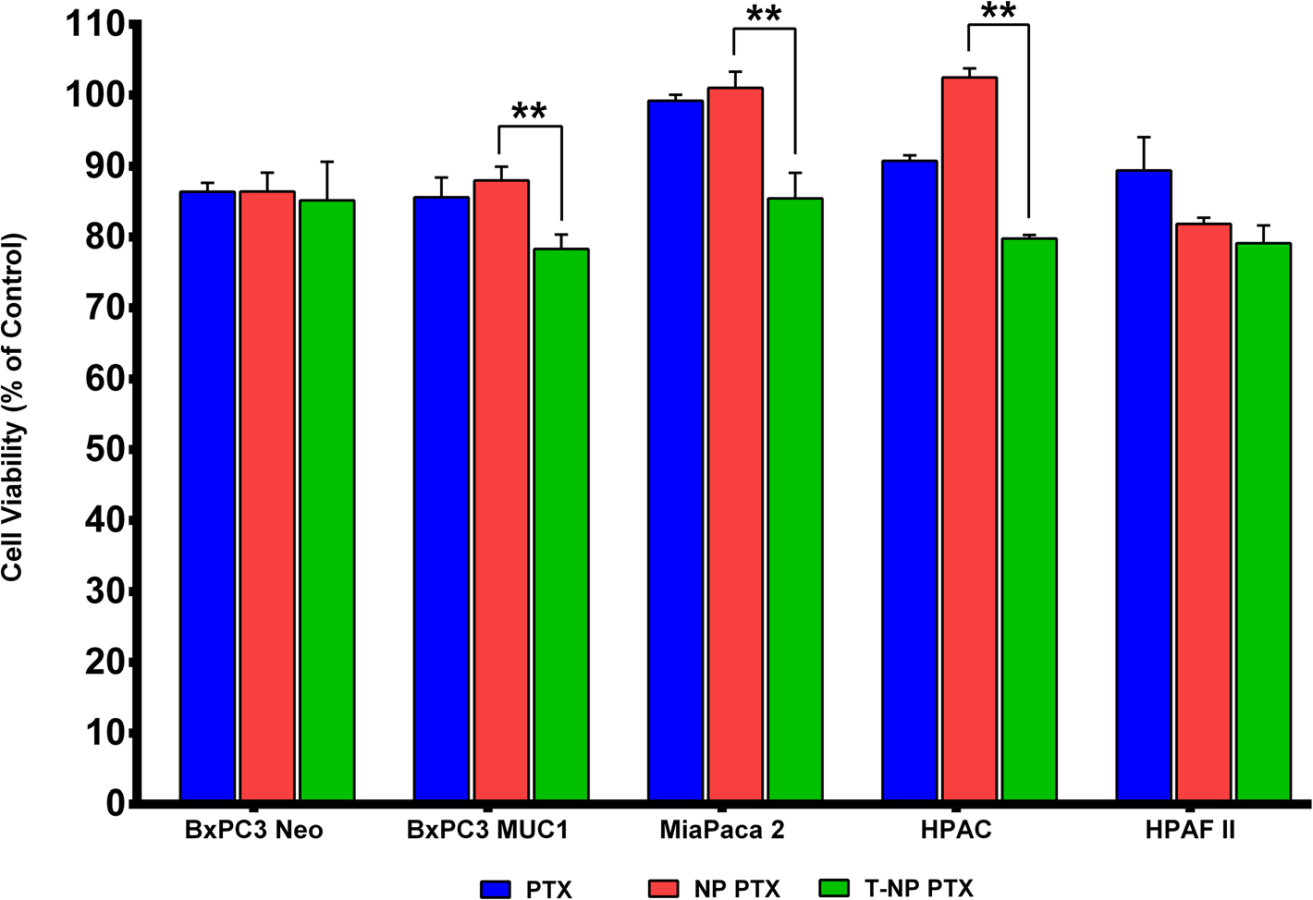
Cell viability of PDA cell lines treated with PTX, PTX loaded NPs and PTX loaded TAB004 conjugated NPs. Concentration of PTX is 3.05 × 10^− 3^ μg/ml. Data shown is mean ± SEM (*n* = 3) and determine by a one-sided t-test comparing treatment groups at each concentration, *p < 0.05, **p < 0.01, ***p < 0.001, ****p < 0.000

Therefore, we next determined the cytotoxicity of PTX loaded T-NPs compared to PTX loaded NPs in the same cells (BxPC3.Neo and BxPC3.MUC1) as well as in a panel of other human PDA cell lines with varying levels of MUC1 expression and sensitivity to PTX (Fig. 6). The comparison we were interested in was between the treatment groups (NP and T-NPs) and not necessarily between the various cell lines. We selected a single dose of PTX for each cell line where at least 90% of cells remained viable post PTX treatment (~IC_10_). We determined if there was any added cytotoxic effect of NPs or T-NPs loaded with PTX at the same concentration as the PTX alone. There was no difference in viability between PTX, NP-PTX or T-NP-PTX treated BxPC3.Neo cells (Fig. 6). This was expected based on Fig. 5c where no difference in internalization and retention was observed in BxPC3.Neo cells between NPs and T-NPs. On the other hand, in MUC1-expressing BxPC3 MUC1, MiaPaca2, and HPAC cells, we observed a significant decrease in cell viability between NP-PTX versus T-NP-PTX at a single dose (Fig. 6). This decrease was not noted in HPAF-II cell even though these cells express high levels of MUC1 (Fig. 6). The reasons for the lack of responsiveness to T-NPs in HPAF-II cells are not currently known. Although the effect of T-NPs versus NPs is modest, it is highly significant because of PDA’s high resistance to chemotherapy.

### TAB004 accumulates at the tumor site and its conjugation to PLGA NPs appears to increase their accumulation in an Orthotopic PDA tumor model

**Fig. 7:**
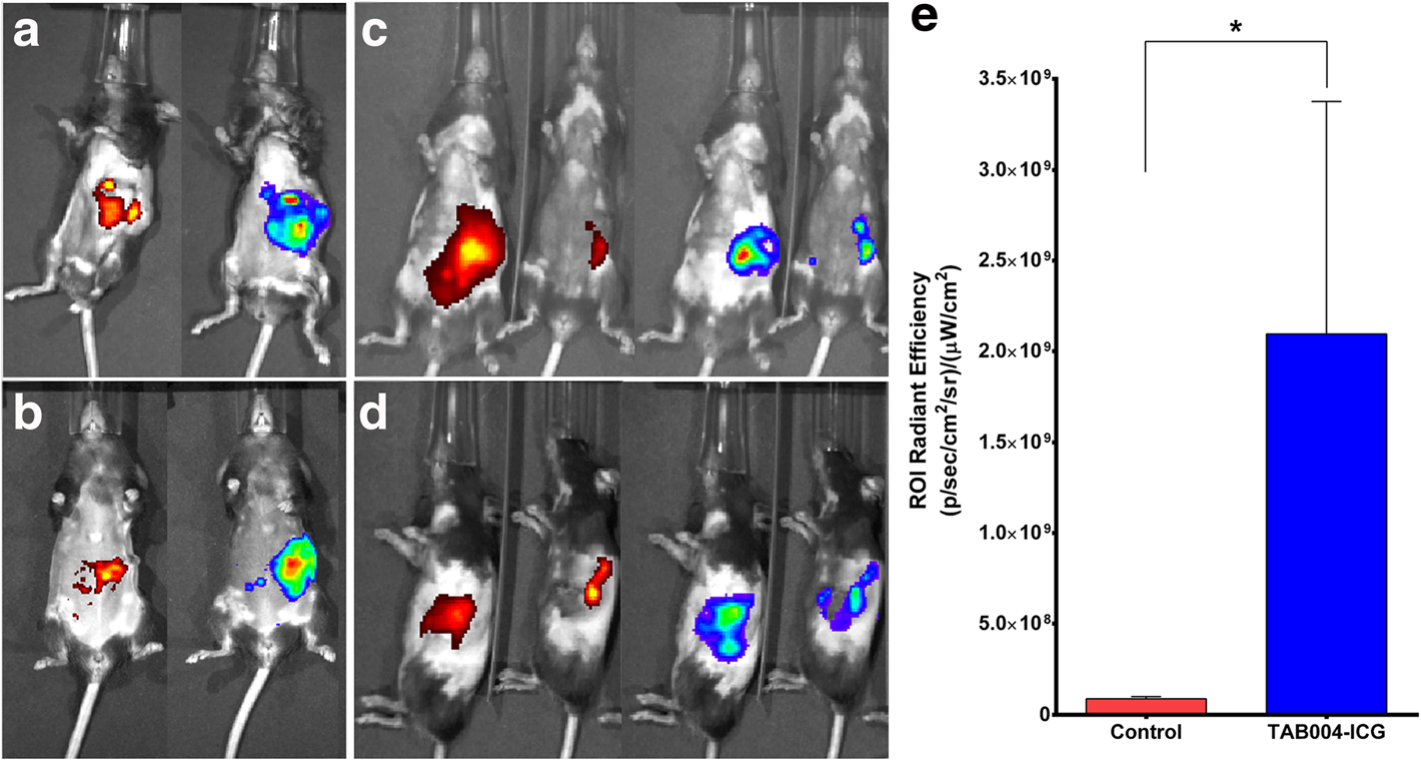
In vivo imaging of TAB004-ICG in orthotopically injected bioluminescent tumor bearing mice (ICG - red/yellow, tumor (luciferase-expressing) – rainbow, *n* = 4): (**a**) mouse 1; (**b**) mouse 2; (**c**) mouse 3 and 4 ventral view; (**d**) mouse 3 and 4 side view; (**e**) quantification of fluorescent radiant efficiency values in TAB004-ICG injected and control mice, *p < 0.05

We demonstrated the specificity of TAB004 in vitro, but the same needed to be determined in vivo. C57BL/6 immune competent mice bearing murine syngeneic orthotopic KCM tumors [35] were injected intraperitoneally with TAB004 conjugated with ICG and imaged 24 h post injection. The KCM cells stably expressed the luciferase gene and thus bioluminescent tumors could be visualized by IVIS post luciferin injection. TAB004 localizes and persists specifically at the tumor site 24 h later (Fig. 7a-d). Images of 4 representative mice are shown. It is clear that the TAB004-ICG localizes only to the bioluminescent pancreatic tumors. The fluorescent radiant efficiency values for region of interests (ROIs) around the tumor site were acquired for TAB004-ICG injected mice and displayed significant increase over tumor bearing control mice that were not injected with TAB004-ICG (Fig. 7e).

Next, we tested ICG loaded NPs and ICG loaded T-NPs in mice bearing the same KCM bioluminescent orthotopic tumors to determine if TAB004 can increase the accumulation of NPs at the tumor site (Additional file 2: Figure S2). ICG loaded NPs appear to clear from the mouse between 24 and 48 h post injection (Additional file 2: Figure S2), similar to the biodistribution profile of ICG loaded NPs injected in non-tumor bearing mice (data not shown). However, ICG loaded T-NPs appeared to accumulate and persist at the tumor site 24 and 48 h post injection (Additional file 2: Figure S2B). Ex vivo images of the tumor and liver of the mice were taken 48 h post injection and ICG loaded T-NPs seem to accumulate and persist in the tumor while ICG load NPs cannot be detected in the tumor post 48 h. We noted that the fluorescence in the liver was identical for ICG loaded T-NPs and ICG loaded NPs (Additional file 2: Figure S2C) suggesting that the tumor localization is extremely high for T-NPs versus NPs. Thus, TAB004 conjugated NPs may be developed as a potential platform for targeted delivery of not only PTX, but other drugs and imaging agents directly to the pancreatic tumor while reducing toxicity to other major organs. Future studies will evaluate the in vivo anti-tumor efficacy in several models of PDA.

## Discussion

The ability to target drug-loaded nanoparticles to the tumor site would greatly enhance efficacy of the drug and reduce toxicity. PLGA is one of the most effective biodegradable polymers used to construct polymeric nanoparticles (NPs). It has been approved by the US FDA for use in drug delivery systems due to controlled and sustained- release properties, low toxicity, and biocompatibility with tissue and cells [42–44]. PEG-functionalized PLGA NPs are especially desirable, as pegylated-NP platforms have demonstrated significantly reduced systemic clearance compared with similar particles without PEG. This design parameter is important for the passive targeting of nanocarrier to tumor by the EPR effects [45]. To enhance tumor-specific targeting, in this study, we aimed to investigate PEG-functionalized PLGA NPs conjugated to monoclonal antibody TAB004. TAB004 specifically recognizes the hypoglycosylated tumor form of MUC1 [32, 46, 47] while sparing recognition of MUC1 on normal epithelial cells. Over 80% of PDA expresses this tumor form of MUC1 and is an established target for immunotherapy [48, 49]. In Fig. 4, we show that TAB004 specifically internalizes in the BxPC3.MUC1 ells but not in BxPC3.Neo cells. Further, we showed that compared to the unconjugated NPs, TAB004 conjugated NPs had significantly enhanced and prolonged cellular accumulation in the BxPC3.MUC1 versus BxPC3.Neo cells confirming antigen specific targeted internalization (Fig. 5). This enhanced cellular internalization and accumulation of T-NPs over NPs is most likely due to the specific binding of TAB004 to tumor form of MUC1 expressed on BxPC3.MUC1 cells thus enabling the NPs to readily internalize through a process of macropinocytosis [50]. Although modest, PTX-loaded T-NPs showed significantly enhanced cytotoxicity (Fig. 6) in an antigen specific manner. The modest enhancement of cytotoxicity may be attributed to the limited time (1.5 h) of exposure of cells to the drug. Longer incubation with the nanoparticles caused degradation of the NPs, which then interfered with the OD values in the survival assay. It is well established that the antitumor effect of PTX results from its intracellular accumulation over time [51]. In vivo in an immune compromised mouse model, we observed specific localization and accumulation of TAB004 only to the orthotropic BxPC3.MUC1 tumors generated in the pancreas (Fig. 7) but not in MUC1-negative tumors or in normal epithelial organs [34]. In a pilot in vivo experiment using immune competent mice, we showed that compared to ICG-NPs, TAB004 conjugated ICG-NPs accumulated in the KCM tumor while unconjugated ICG-NPs failed to accumulate in the tumor (Additional file 2: Figure S2). Thus, we believe that the modest cytotoxic advantage observed in vitro will be significantly enhanced in vivo. Future studies will evaluate the in vivo efficacy of various drug loaded TAB004-NPs in several PDA models. Taken together the data validates the tumor specificity of TAB004 and that loaded NPs conjugated to TAB004 may be a promising nanocarrier for targeted therapy and imaging of PDA.

## Conclusion

Conjugation of NPs to TAB004 greatly enhanced the internalization, retention, and targeting ability of NPs in vitro and in vivo in orthotopic models of human and mouse PDA. TAB004 conjugated PTX loaded NPs showed modest but significant increase in cytotoxicity against PDA cells in vitro. The anti-tumor efficacy of chemotherapeutic drugs in vivo will need to be investigated using this delivery platform.

## Additional files

Additional file 1: **Figure S1.** Confirmation of TAB004 conjugation to PLGA NPs with FACS. Blank NPs were treated with: (A) control (red), TAB004 (blue), anti-mouse IgG_1_ FITC (green), and both TAB004 and anti-mouse IgG_1_ FITC (orange/yellow); (B) control (red), NHS Ester linking reagent (blue), and NHS Ester linking reagent,TAB004, and anti-mouse IgG_1_ FITC (green). (JPG 135 kb)

Additional file 2: **Figure S2.** In vivo imaging of ICG loaded NPs and ICG loaded T-NPs orthotopically injected bioluminescent tumor bearing mice (ICG - red/yellow, tumor – rainbow, *n* = 3): (A) ICG loaded NPs injected into tumor bearing mouse; (B) ICG loaded T-NPs injected into tumor bearing mouse; (C) ex vivo imaging of liver and tumor from (A) and (B). (JPG 173 kb)

## Abbreviations

ACN: Acetonitrile
CDI: 1,1′-carbonyldiimidazole
DAP: 1,3-diaminopropane
DCM: Dry methylene chloride
DMSO: Dimethyl sulfoxide
EE: Encapsulation efficiency
EPR: Enhanced permeability and retention effect
FACS: Fluorescence-activated cell sorting
FBS: Fetal bovine serum
FDA: Fluorescein diacetate
FOLFIRINOX: 5-fluorouracil, oxaliplatin, irinotecan, and leucovorin
HD: Hydrodyamic diameter
ICG: Indocyanine green
IVIS: In vivo imaging system
MTT: 3-(4,5-dimethyathiazol-2-yl)-2,5diphenyltetrazolium bromide
MUC1: Mucin-1
NPs: Nanoparticles
NR: Nile Red
OD: Optical density
PBS: Phosphate-buffered salin
PCL: Polycaprolactone
PDA: Pancreatic ductal adenocarcinoma
PDI: Polydispersity index
PEG: Polyethylene glycol
PLGA: Poly(lactic-co-glycolic acid)
PTX: Paclitaxel
PVA: Poly(vinyl alchohol)
SEM: Scanning electron microscopy
THF: Tetrahydrofuran (oxolane)
tMUC1: Tumor-associated MUC1
T-NPs: TAB004 conjugated to PLGA NPs
ZP: Zeta potential
