# Supplementary figures and images for: Treatment of pancreatic ductal adenocarcinoma with tumor antigen specific-targeted delivery of paclitaxel loaded PLGA nanoparticles
Source: BMC Cancer. 2018 Apr 23;18:457. doi: 10.1186/s12885-018-4393-7 (PMC5914049; doi:10.1186/s12885-018-4393-7)

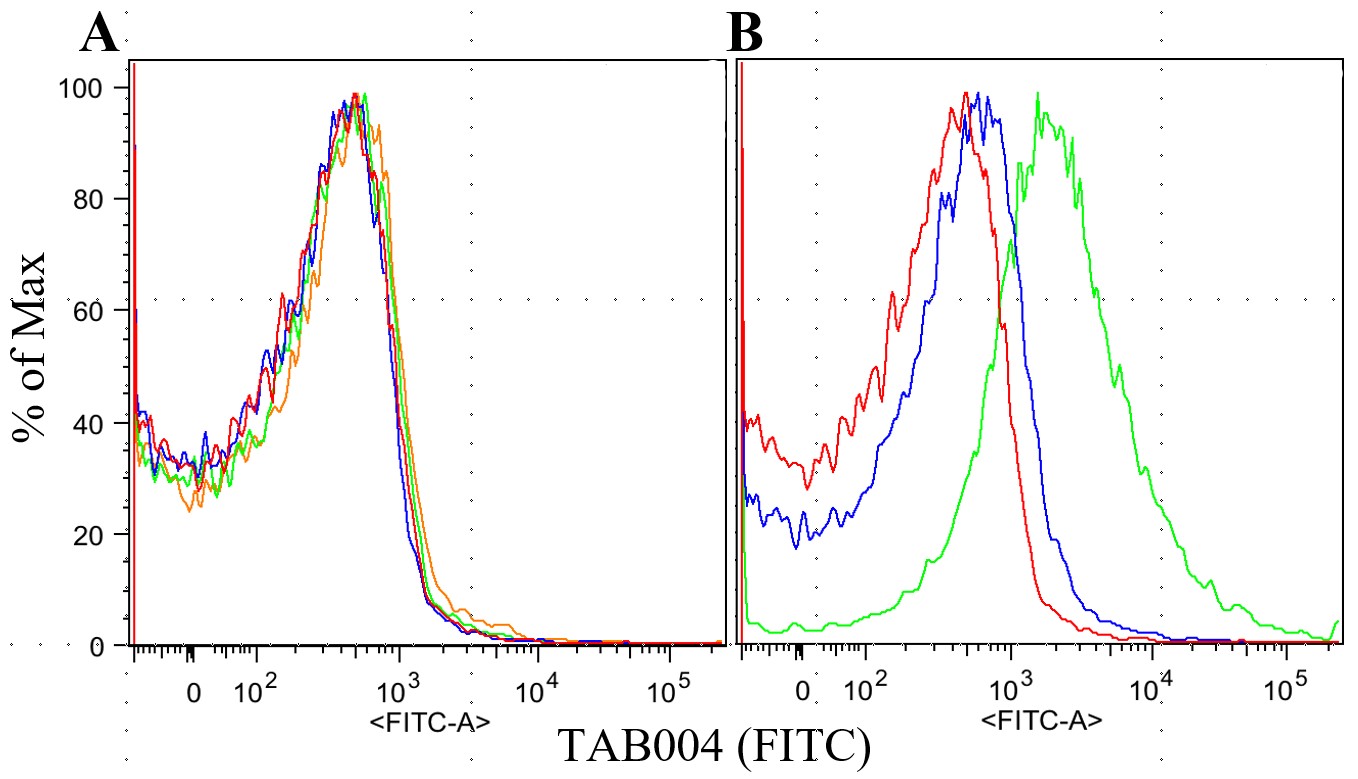

Supplement: Supplementary file 1 — Figure S1. Confirmation of TAB004 conjugation to PLGA NPs with FACS. Blank NPs were treated with: (A) control (red), TAB004 (blue), anti-mouse IgG1 FITC (green), and both TAB004 and anti-mouse IgG1 FITC (orange/yellow); (B) control (red), NHS Ester linking reagent (blue), and NHS Ester linking reagent,TAB004, and anti-mouse IgG1 FITC (green). (JPG 135 kb) [file 12885_2018_4393_MOESM1_ESM.jpg]

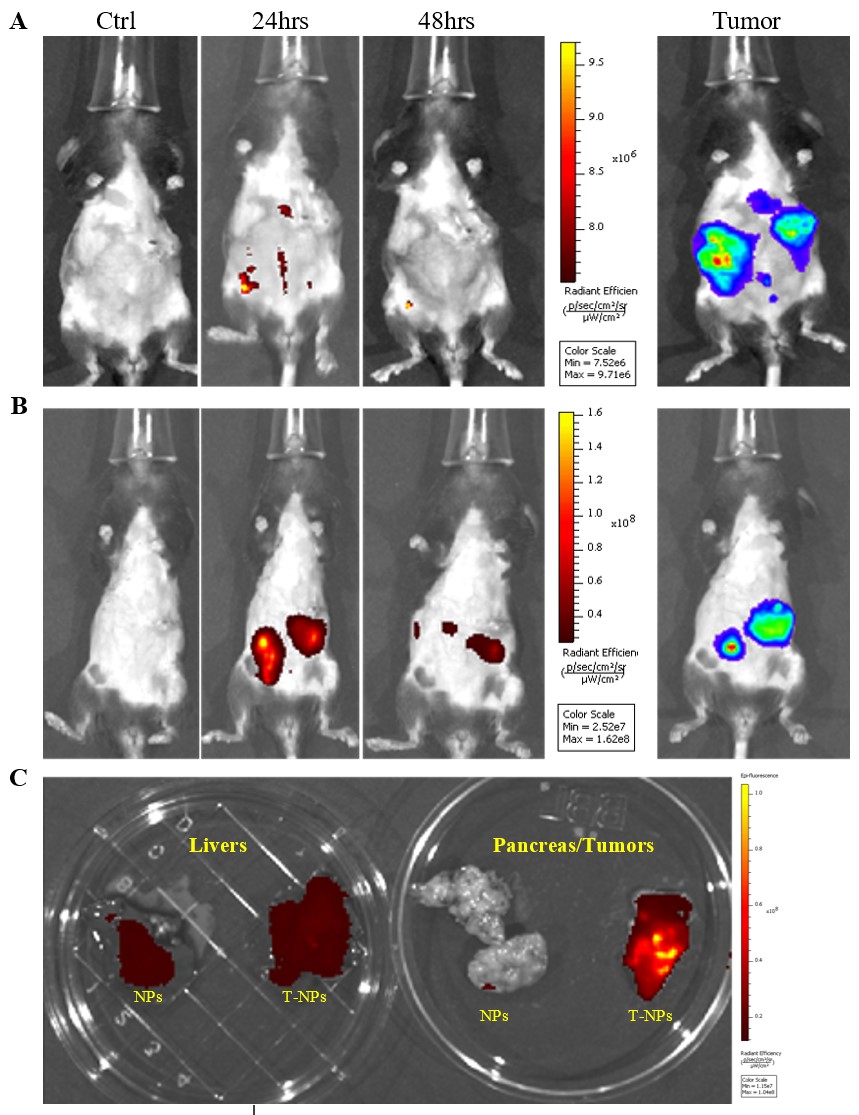

Supplement: Supplementary file 2 — Figure S2. In vivo imaging of ICG loaded NPs and ICG loaded T-NPs orthotopically injected bioluminescent tumor bearing mice (ICG - red/yellow, tumor – rainbow, n = 3): (A) ICG loaded NPs injected into tumor bearing mouse; (B) ICG loaded T-NPs injected into tumor bearing mouse; (C) ex vivo imaging of liver and tumor from (A) and (B). (JPG 173 kb) [file 12885_2018_4393_MOESM2_ESM.jpg]
